# Oral immunization of mice with recombinant *Lactobacillus plantarum* expressing a *Trichinella spiralis* galectin induces an immune protection against larval challenge

**DOI:** 10.1186/s13071-022-05597-w

**Published:** 2022-12-20

**Authors:** Yang Xiu Yue Xu, Xin Zhuo Zhang, Min Min Weng, Yong Kang Cheng, Ruo Dan Liu, Shao Rong Long, Zhong Quan Wang, Jing Cui

**Affiliations:** Department of Parasitology, Medical College, Zhengzhou University, Zhengzhou, 450052 China

**Keywords:** *Trichinella spiralis*, Galectin, *Lactobacillus plantarum* NC8, Protective immunity

## Abstract

**Background:**

*Trichinella spiralis* is an important foodborne parasite that presents a severe threat to food safety. The development of an anti-*Trichinella* vaccine is an important step towards controlling *Trichinella* infection in food animals and thus ensure meat safety. *Trichinella spiralis* galectin (Tsgal) is a novel protein that has been identified on the surface of this nematode. Recombinant Tsgal (rTsgal) was found to participate in larval invasion of intestinal epithelium cells (IECs), whereas anti-rTsgal antibodies impeded the invasion.

**Methods:**

The rTsgal/pSIP409- pgsA′ plasmid was constructed and transferred into *Lactobacillus plantarum* strain NC8, following which the in vitro biological properties of rTsgal/NC8 were determined. Five groups of mice were orally immunized three times, with a 2-week interval between immunizations, with recombinant NC8-Tsgal, recombinant NC8-Tsgal + α-lactose, empty NC8, α-lactose only or phosphate-buffered saline (PBS), respectively. The vaccinated mice were infected orally with *T. spiralis* larvae 2 weeks following the last vaccination. Systemic and intestinal local mucosal immune responses and protection were also assessed, as were pathological changes in murine intestine and skeletal muscle.

**Results:**

rTsgal was expressed on the surface of NC8-Tsgal. Oral immunization of mice with rTsgal vaccine induced specific forms of serum immunoglobulin G (IgG), namely IgG1/IgG2a, as well as IgA and gut mucosal secretion IgA (sIgA). The levels of interferon gamma and interleukin-4 secreted by cells of the spleen, mesenteric lymph nodes, Peyer's patches and intestinal lamina propria were significantly elevated at 2–6 weeks after immunization, and continued to rise following challenge. Immunization of mice with the oral rTsgal vaccine produced a significant immune protection against *T. spiralis* challenge, as demonstrated by a 57.28% reduction in the intestinal adult worm burden and a 53.30% reduction in muscle larval burden, compared to the PBS control group. Immunization with oral rTsgal vaccine also ameliorated intestinal inflammation, as demonstrated by a distinct reduction in the number of gut epithelial goblet cells and mucin 2 expression level in *T. spiralis-*infected mice. Oral administration of lactose alone also reduced adult worm and larval burdens and relieved partially inflammation of intestine and muscles.

**Conclusions:**

Immunization with oral rTsgal vaccine triggered an obvious gut local mucosal sIgA response and specific systemic Th1/Th2 immune response, as well as an evident protective immunity against *T. spiralis* challenge. Oral rTsgal vaccine provided a prospective approach for control of *T. spiralis* infection.

**Graphical Abstract:**

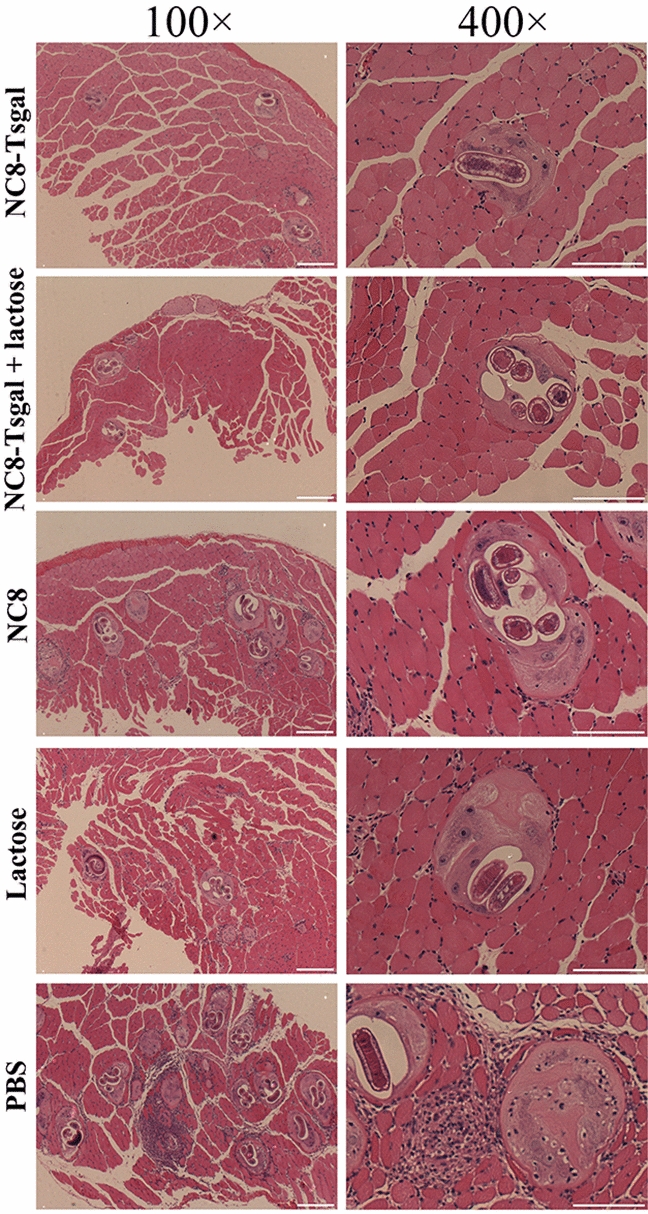

**Supplementary Information:**

The online version contains supplementary material available at 10.1186/s13071-022-05597-w.

## Background

Trichinellosis is an important meat-borne parasitic disease that occurs worldwide [1]. Human *Trichinella* infection is caused by ingesting raw or semi-cooked meats from pigs and other animals infected with *Trichinella* muscle larvae (ML) [2, 3]. Pork from domestic pigs is the primary source of human trichinellosis outbreaks [4, 5]. During 2009–2020, eight outbreaks of human trichinellosis, with 479 cases and two deaths, were documented in China; of these eight outbreaks, seven (87.50%) were caused by eating raw or poorly cooked pork [6]. It is therefore necessary to develop an anti-*Trichinella* vaccine to interrupt the transmission of *Trichinella* infection in food animals and to eliminate this nematode from meat destined for human consumption [7, 8].

Once *Trichinella spiralis*-infected meat has been ingested, digestion of the infected meat by gastric fluids in the stomach liberate the ML from the capsules. These then develop into intestine infectious larvae (IIL) following activation by bile and enteral contents. The IIL penetrate into the intestinal epithelium where they undergo four molts to develop into adult worms (AW) [9, 10]. After copulation, the female adult worms shed newborn larvae (NBL), which enter the venules and lymphatic vessels, spreading throughout the body via blood circulation until they reach the final parasitizing skeletal muscles and develop into the encapsulated ML to finish their life-cycle [11]. Intestinal mucosal epithelium is the first natural defense barrier against intrusion by *T. spiralis* IIL, and it is also the preferential interaction location between the intestinal parasite and the host [12, 13]. The successful IIL invasion of intestinal epithelial cells (IECs) is the key to infection of the host [14, 15]. Therefore, the gut mucosal immune response is crucial for the development of anti-*Trichinella* vaccines [16]. The ideal vaccines should be capable of blocking IIL invasion of the gut epithelium, interrupting IIL development to adulthood, expelling residual IIL and adults from the gut, impeding the deposition of NBL from adult females and killing the escaped NBL and encapsulated larvae in skeletal muscles [17, 18].

Lactic acid bacteria (LAB) belong to the *Clostridium* branch of Gram-positive bacteria. LAB are a group of Gram-positive bacteria that produce lactic acid as the major end product. They include members of the genera *Lactococcus, Lactobacillus* and* Bifidobacterium*, which are commonly found in dairy fermented foods, the environment and animal guts [19]. They have no endotoxin and play an important function in health, facilitating the metabolism of nutrients and dietary polysaccharides, regulating energy balance and initiating and modulating immune responses [20]. *Lactobacillus* has the ability to colonize, express and secrete exogenous antigenic proteins in local gut mucosa, at food-grade safety, and is the preferred bacteria for vaccine carrier development. The expressed and secreted proteins of *Lactobacillus* can stimulate a host’s gut mucosal immune response and generate the corresponding antibodies [21]. Therefore, LAB can be used as a good carrier for the construction of an immune protective vaccine against *Trichinella* infection [22].

In our previous studies, a *T. spiralis* beta-galactoside-binding lectin (galectin, Tsgal; GenBank: XM_003381608.1) was cloned and expressed. The recombinant Tsgal (rTsgal) specially bound to the IECs and mediated *T. spiralis* invasion of the IECs, whereas anti-rTsgal antibodies impeded the invasion of the larvae. Moreover, α-lactose played a suppressive role on rTsgal agglutinating functions [23]. The anchored expression vector pSIP409-pgsA′ contains a pgsA′ protein anchoring sequence for attaching the gene encoding Tsgal, which has been reported to be expressed on the surface of a probiotic recombinant *Lactobacillus plantarum* strain [24]. Our previous studies showed that Tsgal is expressed at all *T. spiralis* developmental stages, and primarily distributed on the surfaces, cuticles, stichosomes and embryos of this nematode [23]. The stichosome consists of a series of stichocytes, with each stichocyte containing many secretory granules that show high antigenicity. Since the surface and excretory/secretory (ES) proteins of *T. spiralis* IIL and AW are the first to be exposed to host’s intestinal mucosa and immune system, they can elicit host’s gut local mucosal and systemic immune response, and produce protective immunity [16]. In the present study, to improve the immune response, we fused recombinant *L. plantarum* expressing Tsgal on its surface to pgsA′ as the protein anchoring sequence and used this product as the Tsgal vaccine candidate. Specific humoral and cellular immune responses and protective immunity against *T. spiralis* infection were investigated in experiments involving oral immunization with the recombinant *L. plantarum* expressing Tsgal in a murine model.

## Methods

### Parasites and experimental animals

The *T. spiralis* isolate (ISS534) used in this study was acquired from a naturally infected domestic pig in central China and passaged in mice in our department, and 4-week-old female BALB/c mice were purchased from Henan Provincial Experimental Animal Center (Zhengzhou, China). The sample size calculation was performed using online software (Experimental design assistant; https://eda.nc3rs.org.uk/eda/login/auth) based on The ARRIVE Guidelines 2.0. The sample size was estimated based on the significance level of 0.05 and power of 0.9. To determine gut mucosal secretion of IgA (sIgA) and cytokine responses, five animals of each experimental group were euthanized at weeks 0, 2, 4, 6, 7 and 11 after vaccination. An additional 10 animals of each group were sacrificed at weeks 7 and 11 after vaccination (e.g. 7 and 35 days post infection [dpi]) to collect enteral adult worms and muscle larvae, respectively. Each experimental group consisted of 50 mice, and 250 mice in total were used in the current study. *Lactobacillus plantarum* NC8 was a gift from Professor Gui Liang Yang (College of Animal Science and Technology, Jilin Agricultural University, China [22]) and maintained in our laboratory [24].

### Preparation of rTsgal and anti-rTsgal serum

The complete functional domain of the Tsgal gene (XM_003381608.1), containing 795 bp encoding 244 amino acids, was cloned, and recombinant plasmid pQE-80L/Tsgal was transferred into *Escherichia coli* strain BL21 (DE3) (Novagen, Pledran, France). The expression of rTsgal protein was induced using 0.5 mM isopropyl β-d-1-thiogalactopyranoside (IPTG) for 6 h at 37 °C [25]. The rTsgal protein was first purified and then treated with High Capacity Endotoxin Removal Resin (Pierce™, Thermo Fischer Scientific, Waltham, MA USA) as previously described [26]. Twenty female mice were subcutaneously immunized using 20 μg rTsgal emulsified in complete Freund’s adjuvant. Two booster immunizations were administered with 20 μg rTsgal emulsified in incomplete Freund’s adjuvant at a 14-day interval [27]. At 2 weeks following the third immunization, tail blood was collected, and anti-rTsgal serum was isolated and stored at - 80 °C until use [28]. Serum was also collected from 10 mice experimentally infected with 300 T*. spiralis* ML at 35 dpi (infection serum), and normal serum was obtained from 10 normal mice before immunization.

### Construction of recombinant* L. plantarum *NC8-Tsgal

The functional domain of the Tsgal gene was amplified by PCR with specific primers carrying *Xbal*I and *Hind*III (bold and shaded) were selected as restriction sites to design Tsgal specific primers (5′-CGCAAAGTTCCGTATTTAGCCAAGTTGG-3′, 5′-CGC TCATTCTAAATGAATCAACTGC-3′). The amplified Tsgal DNA fragment was cloned into expression vector pSIP409-pgsA′. The recombinant pSIP409-pgsA′-Tsgal was transferred into *L. plantarum* NC8 by electroporation, and successful construction of the recombinant NC8-Tsgal strain was verified by PCR. In order to evaluate the effect of pSIP409-pgsA′-Tsgal on the growth of *L. plantarum*, the recombinant NC8-Tsgal was cultured in MRS broth culture medium at 30 °C for 24 h, and the optical density (OD) of bacterium solution was measured by ultraviolet spectrophotometry at 600 nm (OD_600nm_) every 2 h during the cultivation; empty NC8 was used as the control. Moreover, to observe the survival of NC8-Tsgal under various pH conditions, the in vitro gastric environment with diverse pH values was simulated, as previously described [29].

### Immunofluorescence test

To assess the Tsgal expression on the surface of NC8-Tsgal, we performed the immunofluorescence test (IFT) as reported previously [30]. Briefly, NC8-Tsgal was first blocked using 1% bovine serum albumin (BSA; Aldrich-Sigma, St. Louis, MO, USA). After washes in PBS, NC8-Tsgal was probed at 37 °C for 2 h with anti-rTsgal serum, infection serum and normal serum (1:100). Following further washes in PBS, NC8-Tsgal was incubated with cy3/FITC-anti-mouse immunoglobulin G (IgG) conjugate (1:100; Santa Cruz Biotechnology, Dallas, TX, USA) and then observed under a fluorescence microscope (Olympus, Tokyo, Japan) [31, 32].

### Western blotting analysis

The NC8-Tsgal was cultured in MRS medium containing 10 μg/ml erythromycin to an OD_600nm_ of 0.3 at 30 °C, and then 50 ng/ml SppIP (sakacin P) was added to induce the expression of Tsgal [22]. Western blotting was performed to detect Tsgal expression in NC8-Tsgal, as reported previously [33, 34]. Soluble proteins of NC8-Tsgal were transferred to a nitrocellulose membrane (MilliporeSigma, Burlington, MA, USA). The membrane was blocked with 5% skim milk at 37 °C for 2 h, cut into strips and incubated with anti-rTsgal serum, *T. spiralis*-infected mouse serum and pre-immune normal mouse serum (1:100). After washing with Tris-bufered saline containing Tween (TBST), the strips were incubated with horseradish peroxidase (HRP)-anti-mouse IgG conjugate (1:10,000; SouthernBiotech, Birmingham, AL, USA) and finally stained with 3-amino-9-ethylcarbazole (AEC; Solarbio, Beijing, China) [35, 36].

### Immunization of BALB/c mice with NC8-Tsgal

To investigate the protective effect of rTsgal and α-lactose on *T. spiralis* challenge infection, NC8-Tsgal/α-lactose was used to inoculate the mice. The α-lactose (alpha-d-glucopyranose) was purchased from Kemiou (Tianjin, China; purity = 99%). The α-lactose was dissolved in sterile water (277 mM), and the resulting solution filtered through a 0.22-μm filter.

The 250 mice included in this study were randomly divided into five groups (50 animals per group): (i) NC8-Tsgal group; (ii) NC8-Tsgal + α-lactose group; (iii) NC8 control group; (iv) α-lactose control group; and (v) PBS control group. The concentration of the bacterial suspension was adjusted to 1 × 10^10^ CFU/ml. The NC8-Tsgal and NC8 empty bacteria groups were orally administered 200 μl of the corresponding bacterial solution once daily for 3 days, and the PBS group was given an equal volume of PBS. Two booster immunizations were given as the same dosage of NC8-Tsgal at an interval of 2 weeks. The NC8-Tsgal + α-lactose group received the NC8-Tsgal vaccination at the same time as the NC8-Tsgal group, but the former group was also orally administered with 200 μl of the 277 mM α-lactose solution twice daily for 2 weeks beginning at 1 week before challenge to 1 week after the challenge. The α-lactose control group received an equal dose of α-lactose at the same time as the NC8-Tsgal + α-lactose group received the NC8-Tsgal vaccination.

**Fig. 1:**
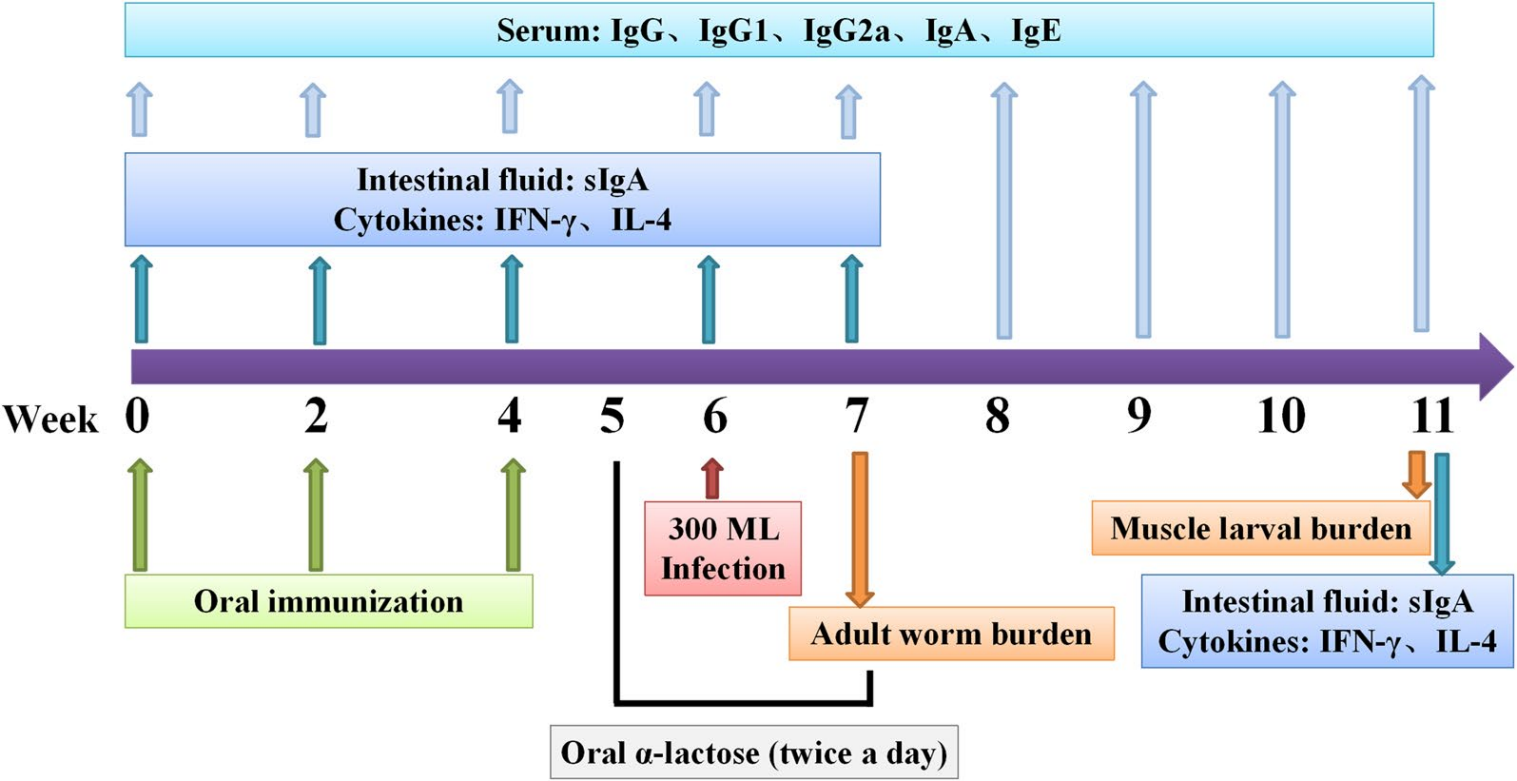
The designed immunization scheme and assay protocol. A total of three oral immunizations were given, and five mice of each group were euthanized for assays of intestinal sIgA and cytokines at weeks 0, 2, 4, 6, 7 and 11 after the first immunization. Serum-specific anti-*Trichinella spiralis* galectin (Tsgal) antibodies (total IgG, IgG1 and IgG2a) were measured by indirect enzyme-linked immunosorbent assay [ELISA] using recombinant Tsgal (rTsgal) at 2 weeks following each immunization and at weeks 1–5, respectively, following challenge. After being challenged with 300 *T. spiralis* muscle larvae, 10 mice of each group were sacrificed at weeks 7 and 11 after vaccination (i.e. 7 and 35 days post infection [dpi), and adults worms and muscle larvae were recovered to assess the protective efficacy of recombinant NC8-Tsgal/α-lactose against *T. spiralis* challenge. Histopathological changes of the intestine and muscles from infected mice were examined at 1 and 5 weeks after challenge (i.e. 7 and 35 dpi). Samples were collected at the time-points indicated in the figure. Ig, Immunoglobulin; IL, interleukin; IFN, interferon; sIgA, secretory (mucosal) IgA

At weeks 0, 2, 4, 6 and 7–11 after the first vaccination, 100 μl of blood was collected from the tail tip of 10 mice in each group, and serum samples were collected and preserved at - 80 °C until use [37]. Five mice from each group were euthanized at 0, 2, 4, 6, 7 and 11 weeks after the first vaccination, and the intestine, spleen, mesenteric lymph nodes (MLN), Peyer's patches (PP) and intestinal lamina propria (ILP) cells were collected at the respective time-points [33, 38]. To evaluate vaccine efficacy, 10 mice of each group were euthanized at weeks 7 and 11 after vaccination (i.e. 7 and 35 dpi), and intestinal adult worm and ML burden (larvae per gram [LPG]) were measured. The scheme of the immunization protocol is shown in Fig. 1.

### Detection of serum anti-rTsgal antibodies by enzyme-linked immunosorbent assay

Serum-specific IgG, IgG1, IgG2a and IgA levels in all immunized mice were determined by enzyme-linked immunosorbent assay (ELISA) using rTsgal as coating antigen [39]. In brief, the plate was coated with 2 μg/ml rTsgal at 4 °C overnight, and then the plate was blocked with 5% skimmed milk in PBS with Tween (PBST) for 2 h at 37 °C. After washing in PBST, the plate was probed with 1:100 dilutions of the various sera for 1 h at 37 °C, and then incubated with HRP-anti-mouse IgG conjugate (1:10,000; Southern Biotech) at 37 °C for 1 h. Following further washes,* o*-phenylenediamine dihydrochloride (OPD; Alrich-Sigma) was used as the substrate for coloration; OD values were measured at 492 nm with a microplate reader (Tecan, Schweiz, Switzerland) [11, 40].

### Assessment of enteral sIgA and histamine

To assess total and Tsgal-specific sIgA in the gut fluids, washed gut was recovered as described previously [7, 41]. In brief, a 20-cm-long intestinal segment was cut out, and the gut interior was washed 3 times with 1 ml of cold PBS with 1% protease inhibitor (Sangon Biotech, Shanghai, China). The washing fluid was recovered and then centrifuged at 12,000 *g* for 5 min at 4 °C to eliminate fecal matter and/or tissue debris. Total gut sIgA was measured by a sandwich ELISA as previously reported [16, 42]. Tsgal-specific sIgA was determined by ELISA with 2 μg/ml of rTsgal. Coloration was developed with OPD (Aldrich-Sigma) plus H_2_O_2_, the reaction was stopped with 2 M H_2_SO_4_. The OD values at 492 nm were measured using a microplate reader (Tecan) [43, 44].

As the histamine secreted by gut epithelial mast cells has an obvious effect on intestinal inflammation and adult worm expulsion from the gut, histamine levels in gut fluids were assessed at weeks 0, 2, 4 and 6 after vaccination, and at weeks 1 and 5 following larval challenge. The levels of intestinal histamine concentrations were measured using a mouse ELISA kit according to the manufacturer's instructions (Elabscience Biotechnol, Wuhan, China). The data were presented in nanograms per milliliter (ng/ml) ± standard deviation (SD). All testing of samples was carried out in duplicate [45].

### ELISA determination of cytokine response to oral rTsgal vaccination

To examine Tsgal-specific cellular immune responses, five mice of each group were euthanized at weeks 0, 2, 4 and 6 following immunization, and at 1 and 5 weeks after infection. The spleen, MLN, PP and ILP were isolated from immunized mice and homogenized in complete RPMI-1640 medium (g) [46]. The pellets were collected after centrifugation at 300 *g* for 15 min, and the cells were isolated as reported [30]. The cells were adjusted to a density of 5 × 10^6^ cells/ml in RPMI-1640 medium containing 5% fetal bovine serum (FBS; Gibco™, Thermo Fisher Scientific), stimulated with 4 μg/ml rTsgal and incubated for 3 days. After incubation, the levels of interferon gamma (IFN-γ) and interleukin-4 (IL-4) in RPMI-1640 medium were assessed using sandwich ELISA and showed as pictograms per milliliter (pg/ml) [17, 47].

### *Trichinella spiralis *challenge infection and evaluation of immune protection

To assess the immune protection produced by oral NC8-Tsgal, all vaccinated mice were orally infected with 300 *T. spiralis* ML at 2 weeks after the final vaccination. Ten mice from each of the five groups were euthanized at 7 and 35 dpi to recover AW of intestines and ML of skeletal muscles, respectively [15]. The immune protection was ascertained according to mean number of intestinal AW and muscle LPG from NC8-Tsgal immunization group relative to the PBS group [24, 48, 49].

### Histopathological examination of murine intestine and skeletal muscle

At 7 and 35 dpi, small intestine and masseter muscles were collected from three infected mice per group and fixed in 4% formalin for 24 h, embedded in paraffin wax and cut into 2-μm-thick tissue cross-sections; the tissue sections were then deparaffinized and stained with using hematoxylin and eosin (HE) stain and periodic acid-schiff stain (PAS; Baso, Zhuhai, China) [50]. The sections were then observed under light microscopy, and the inflammatory cells (eosinophils, neutrophils and lymphocytes) and goblet cells per field (400×) were examined and counted to assess the pathological change in intestine and muscles, as previously reported [18].

### Quantitative PCR assay of mucin 2 messenger RNA expression in gut epithelium of immunized mice

RNA extraction was performed with TRIzol reagent (Invitrogen™, Thermo Fisher Scientific) by lysing 100 mg of small intestine tissue samples of five infected mice per group at 7 dpi. The Mucin 2 (Muc2) messenger RNA (mRNA) expression level was assessed using quantitative PCR as previously reported [51, 52]. The specific primers of Muc2 were 5′-TGTGGCCTGTGTGGGAACTTT-3′ and 5′-CATAGAGGGCCTGTCCTCAGG-3′. The relative level of Muc2 mRNA expression was normalized by subtracting the mRNA expression level of a murine housekeeping gene (glyceraldehyde-3-phosphate dehydrogenase [GAPDH]; GenBank: NM_001289726.1), and then calculated in line with the comparative Ct (^2−ΔΔCt^) method [9, 53]. Each experiment was carried out in triplicate.

### Statistical analysis

All data were analyzed using SPSS version 21.0 software (SPSS IBM Corp., Armonk, NY, USA), and the results were shown as mean ± SD. The Student’s t-test was used to compare the differences between IgG1 and IgG2 levels of the Tsgal-NC8 group and NC8 + lactose group. One-way analysis of variance (ANOVA) was used to analyze the differences among various groups, then Dunnett’s T3 test (for the AW burdens and ML burdens) and LSD test (for the others) were used as post-hoc tests, respectively. *P* < 0.05 was defined as statistical significance.

## Results

### Construction of NC8-Tsgal

NC8-Tsgal was digested using *Xbal*I and *Hind*III. Electrophoresis of the PCR products showed the successful construction of NC8-Tsgal with an insert of about 795 bp. Sequence analysis indicated that the amplified Tsgal gene fragment consisted of 795 bp and was correctly cloned into the pSIP409-pgsA′, with 99.87% identity to those of the Tsgal sequence in GenBank (XM_003381608.1).

### Biological properties of NC8-Tsgal

Growth curve analysis revealed that the recombinant plasmid pSIP409-pgsA′-Tsgal did not suppress the proliferation of NC8**-**Tsgal, and no significant difference was observed between the growth curve of NC8-Tsgal and the normal empty NC8. The results of the in vitro simulation under conditions of the gastric acid environment showed that NC8-Tsgal survived for 2–3 h in the acidic environment (pH 1.0–2.0) and for a longer time at pH 3.0–4.0. The number of recombinant NC8-Tsgal bacteria was clearly lower in the environment at pH 1.0–4.0 than in that at pH 6.4 (*F* = 275.056, *P* < 0.05) (Additional file 1: Figure S1).

### Expression of Tsgal in NC8-Tsgal

**Fig. 2:**
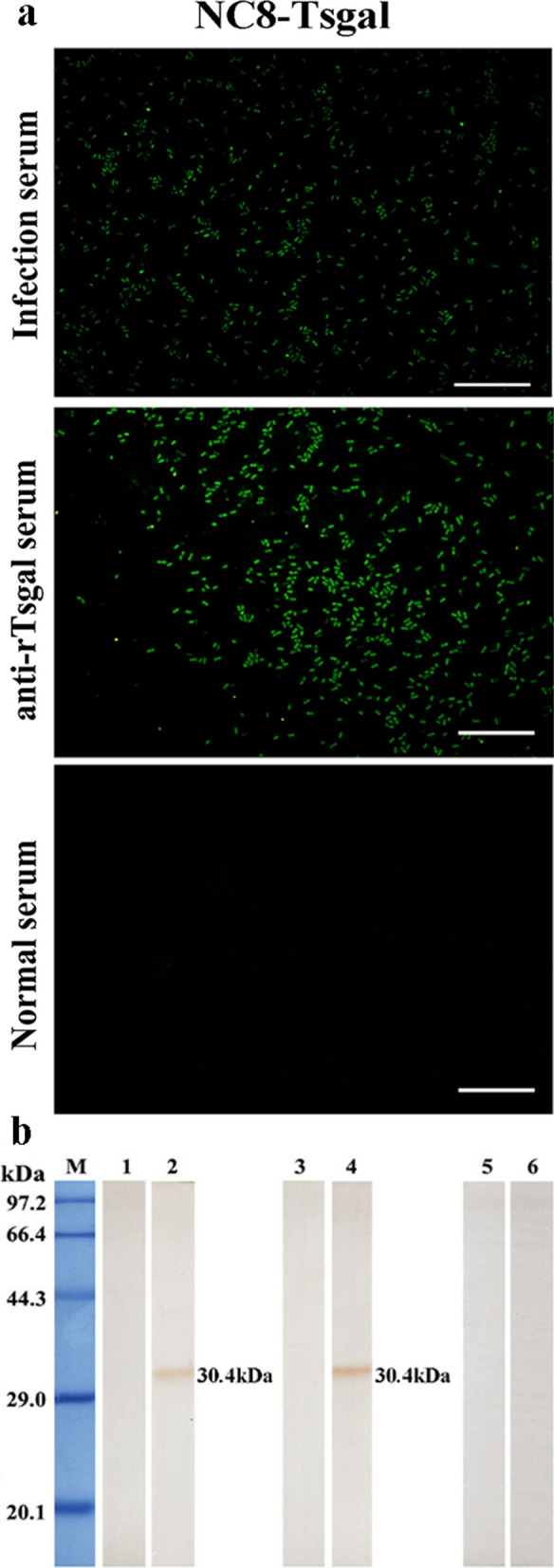
Expression of Tsgal on recombinant NC8-Tsgal bacterium surface by the immunofluorescence test (IFT) and western blotting. **a** Green fluorescence staining was observed on the surface of* Lactobacillus plantarum* strain NC8-Tsgal (NC8-Tsgal) by IFT using anti-rTsgal serum. The bacteria recognized by infection serum (serum collected from mice experimentally infected with T*. spiralis* ML) as a positive control, and normal serum (serum collected from normal mice before immunization) as the negative control. Scale bar: 5 μm. **b** Western blotting analysis of Tsgal expression. Soluble proteins of normal NC8 (lanes 1, 3, 5) and NC8-Tsgal (lanes 2, 4, 6) were probed by infection serum (lanes 1, 2), anti-rTsgal serum (serum from mice immunized with rTsgal; lanes 3, 4) and normal serum (lanes 5, 6), respectively. The expressed and recognized Tsgal bands have a molecular weight of about 30.4 kDa

The IFT revealed positive green fluorescence staining on the surface of NC8-Tsgal using anti-rTsgal serum and infection serum (Fig. 2a). Western blotting results showed that an individual protein band of NC8-Tsgal of about 30.4 kDa was recognized by the anti-rTsgal serum and infection serum, but no bands were identified in soluble proteins of the empty NC8 (Fig. 2b). These results demonstrated that the Tsgal protein was successfully expressed on the surface of NC8-Tsgal.

### Serum anti-Tsgal antibody responses in immunized mice

**Fig. 3:**
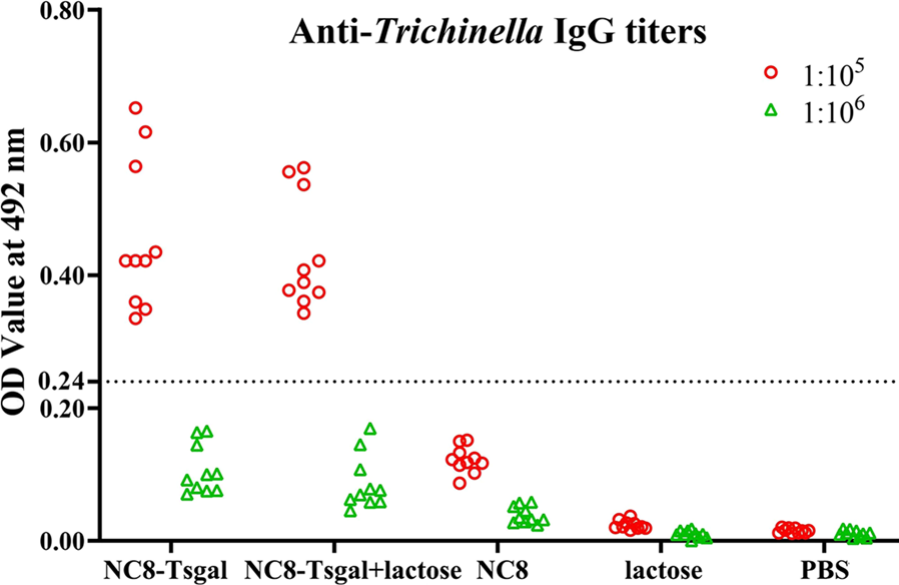
Serum anti-Tsgal IgG measured by ELISA with rTsgal. Anti-Tsgal IgG levels were assayed 2 weeks after the last immunization in sera of mice immunized with NC8-Tsgal. Serum samples diluted at 1:10^5^ and 1:10^6^, respectively, were measured by ELISA with rTsgal. All serum samples were assayed in duplicate. The data are presented as the OD values of anti-Tsgal IgG levels from 10 vaccinated mice. Forty serum samples (1:100 dilutions) from normal mice were measured as negative serum controls. The cut-off value of ELISA was calculated based on the 2.1-fold the mean OD value of the negative control serum from normal mice. Serum OD values that were greater than the cut-off value were regarded as being positive. The cut-off value (0.24) is shown with a dotted line. OD, Optical density; PBS, phosphate-buffered saline

Serum anti-Tsgal antibody IgG titers 2 weeks after the third vaccination were measured by ELISA using rTsgal. Anti-Tsgal IgG levels in vaccinated mice were significantly increased in comparison with pre-vaccination levels (*P* < 0.0001), and mean antibody titer of the recombinant NC8 immunized groups reached 1:10^5^ after the final vaccination, indicating that recombinant NC8 had a good immunogenicity (Fig. 3).

**Fig. 4:**
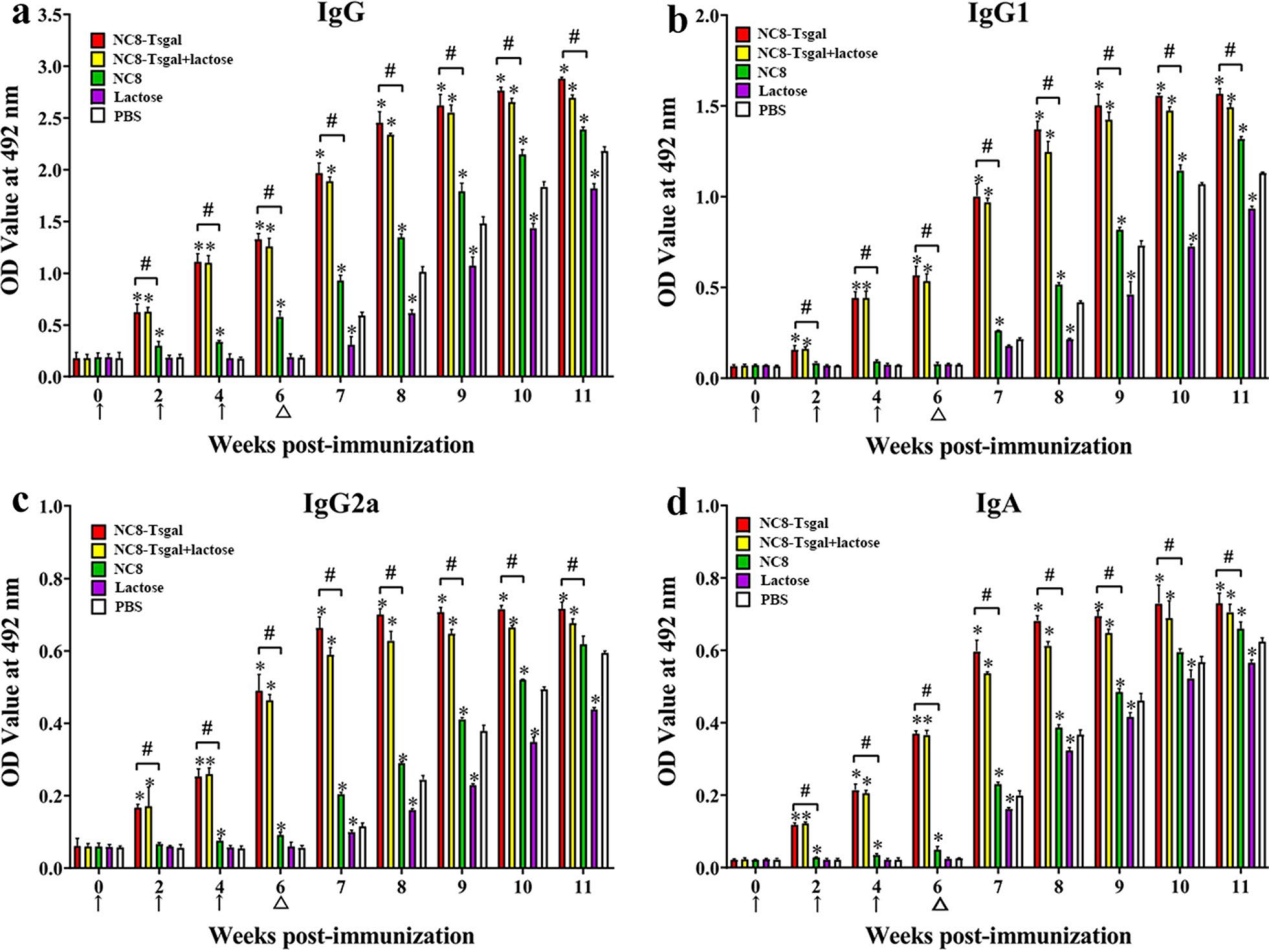
Detection of anti-Tsgal antibodies in mice orally immunized with recombinant *L. plantarum* NC8 by ELISA. All serum samples were tested in duplicate. The data are presented as the mean OD values ± standard deviation (SD) of anti-Tsgal IgG level from 10 vaccinated mice. **a** Serum anti-Tsgal IgG of vaccinated mice at diverse time intervals after immunization was assessed by ELISA.** b**,** c** Specific IgG1 (**b**) and IgG2a (**c**) subclass responses were also ascertained at various times after vaccination. **d** Specific IgA level in vaccinated mice. The vaccination times are shown with arrows (↑) and the time of *T. spiralis* challenge time is indicated by triangles (△). Asterisk (*) indicates significance at *P* < 0.05 compared to the PBS group; hashtag (#) indicates significance at *P* < 0.05 compared to the empty NC8 control group (one-way analysis of variance with LSD test)

The ELISA results showed that the serum anti-Tsgal IgG levels of the NC8-Tsgal and NC8-Tsgal + α-lactose groups at 2 weeks following vaccination were significantly increased in comparison with pre-vaccination levels (*P* < 0.05) and that they continued to increase at 4 and 6 weeks after vaccination and at 1–5 weeks following challenge infection. Tsgal-specific IgG levels of NC8-Tsgal and NC8-Tsgal + α-lactose groups were significantly higher than those of the three control groups at weeks 2, 4 and 6 after vaccination (*P* < 0.0001) (Fig. 4a). Both IgG1 and IgG2a levels were also clearly higher than those of the three control groups at 2 weeks following vaccination (*P* < 0.0001) (Fig. 4b, c). The IgG1 level of the NC8-Tsgal group at 4 and 6 weeks after vaccination was clearly higher than that of IgG2a (*t*_4W_ = 14.923, *t*_6W_ = 3.580, *P* < 0.01). The IgG1 level of the NC8-Tsgal + α-lactose group at 4 and 6 weeks after vaccination was clearly higher than the IgG2a level (*t*_4W_ = 14.212, *t*_6W_ = 5.466, *P* < 0.0001), indicating that NC8-Tsgal immunization triggered a mixed Th1/Th2 immune response with Th2 predominance. Compared to the three control groups, serum IgA levels of the NC8-Tsgal and NC8-Tsgal + α-lactose groups were also significantly increased (*P* < 0.05) (Fig. 4d). Nevertheless, the IgG or IgA levels between the two immunization groups (NC8-Tsgal and NC8-Tsgal + α-lactose) were not significantly different at weeks 2, 4 and 6 after vaccination (IgG: *t*_2W_ = 0.189, *t*_4W_ = 0.333, *t*_6W_ = 2.194, *P *> 0.05; IgA: *t*_2W_ = 1.922, *t*_4W_ = 1.421, *t*_6W_ = 0.745, *P* > 0.05), suggesting that lactose inoculation did not affect and enhance the humoral immune response of immunization of mice with NC8-Tsgal. Moreover, the mice orally inoculated with empty NC8 alone, lactose only or PBS only did not exhibit any anti-Tsgal IgG and IgA responses at weeks 2, 4 and 6 after vaccination; however, after larval challenge, the three control groups also showed increasing serum levels of anti-Tsgal IgG and IgA in comparison with pre-challenge levels (*P* < 0.0001).

### Intestinal mucosal immune response

**Fig. 5:**
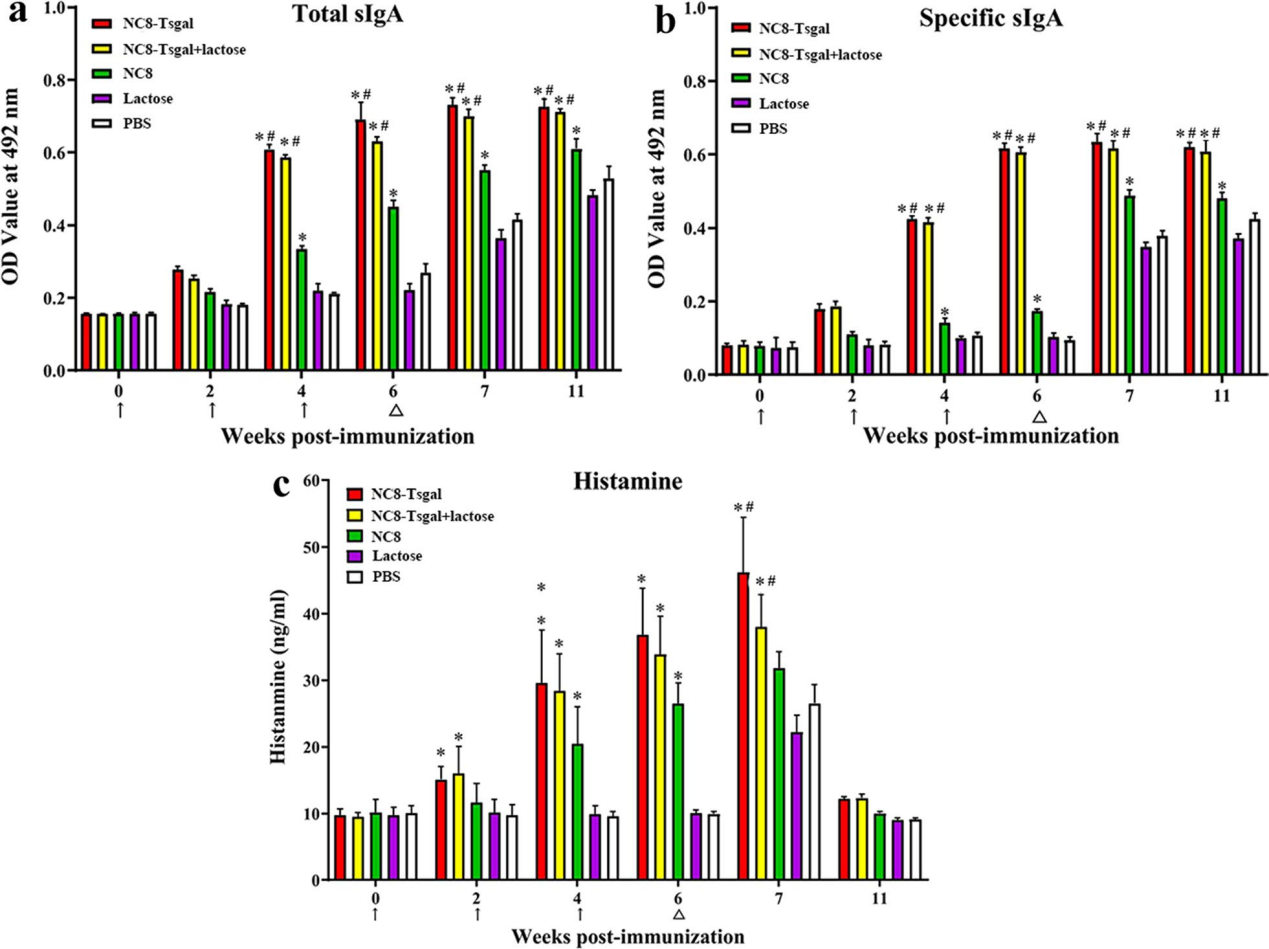
Levels of total sIgA (**a**), Tsgal-specific sIgA (**b**) and histamine (**c**) in enteral washes of immunized mice. The data are shown as the mean values ± SD from 5 animals per group. No evidently detectable sIgA response and histamine secretion was observed in the lactose or PBS control group. The vaccination times are shown with arrows (↑) and the challenge infection time is indicated by triangles (△). Asterisk (*) indicates a significant difference at *P* < 0.05 compared to the lactose or PBS group; hashtag (#) indicates a significant difference at *P* < 0.05 compared to the empty NC8 control group (one-way ANOVA with LSD test)

Statistical comparison of sIgA levels among various groups was performed with one-way ANOVA followed by LSD test. Total sIgA levels of the NC8-Tsgal and NC8-Tsgal + α-lactose groups were significantly higher than those of the three control groups at weeks 4 and 6 after vaccination (*P* < 0.05) (Fig. 5a). The Tsgal-specific sIgA levels of these same two Tsgal immunization groups at weeks 4 and 6 after vaccination was also clearly higher than those of the three control groups (*P* < 0.0001) (Fig. 5b). Also, the higher levels of total and specific sIgA in the two Tsgal immunization groups were maintained up to 5 weeks after the challenge. However, the specific sIgA levels in the two Tsgal immunization groups were not statistically different after vaccination and challenge (*P ˃* 0.05). No specific enteral mucosal sIgA responses were observed in mice orally injected with only empty NC8, only lactose or only PBS.

Histamine concentration of the enteral fluid at various times after immunization and challenge was assayed by ELISA. The results revealed that compared to the lactose or PBS group, the histamine levels in the NC8-Tsgal, NC8-Tsgal + α-lactose and empty NC8 groups were significantly increased at 4 and 6 weeks after the first immunization (*P* < 0.0001), continued to rise at 1 week after *T. spiralis* infection (*P* < 0.05) and regressed at 5 weeks after infection (Fig. 5c). These results suggested that oral immunization of mice with NC8-Tsgal or empty NC8 triggered an intestinal mucosal immune response and histamine secretion.

### ELISA determination of cytokine responses

**Fig. 6:**
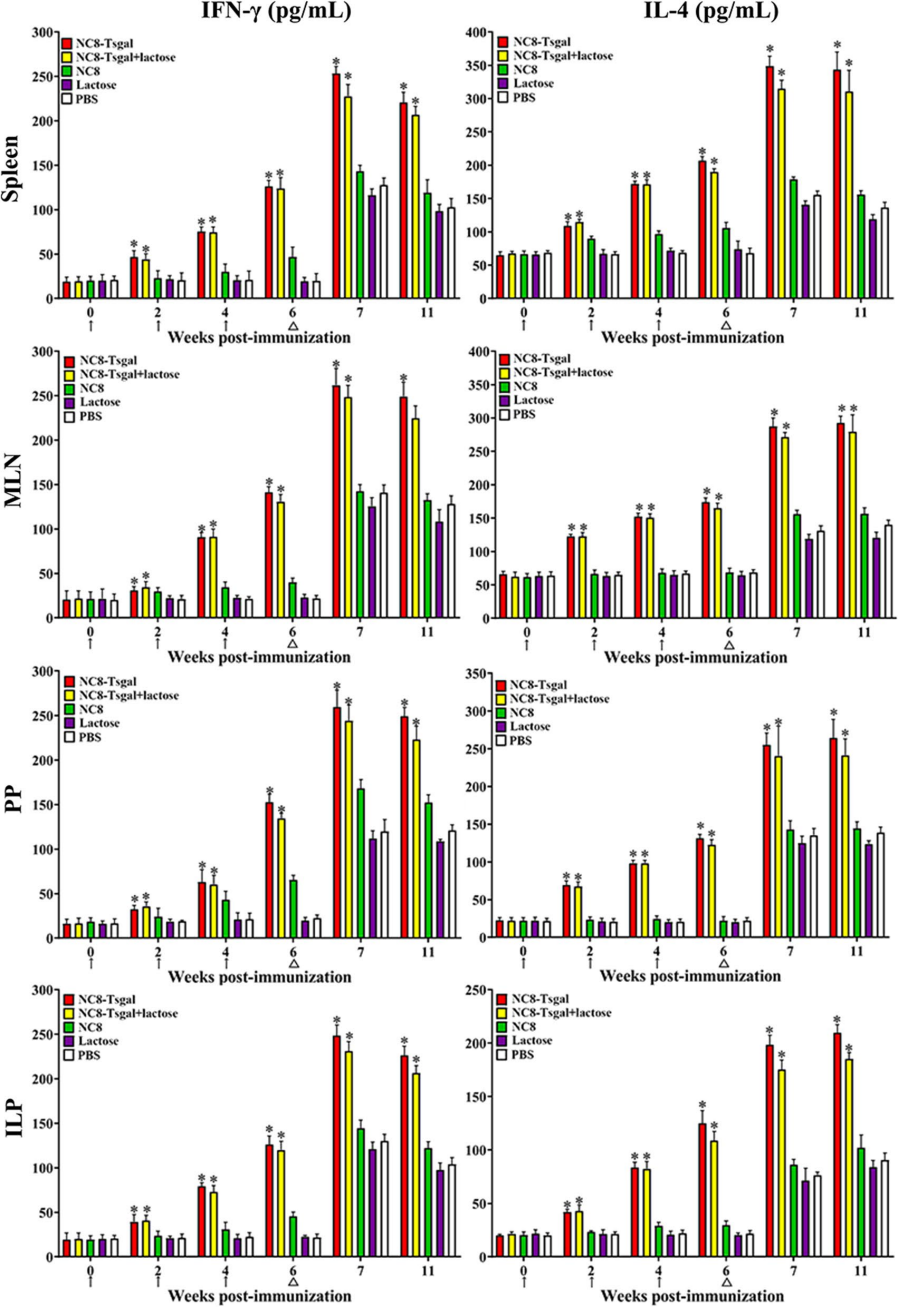
Levels of IFN-γ and IL-4 secreted by spleen, MLN, PP and ILP from immunized mice at different times after vaccination. Concentrations of the two cytokines (IFN-γ and IL-4) were determined in supernatant after the spleen, MLN, PP and ILP cells were stimulated with 4 μg of rTsgal at 37 °C and 5% CO_2_ for 72 h. The data are shown as the mean ± SD of 5 mice per group. The vaccination times are shown with arrows (↑). *T. spiralis* challenge infection time is indicated by triangles (△). Asterisk (*) indicates significance at *P* < 0.0001 compared to the empty NC8, lactose or PBS group (one-way ANOVA with LSD test). ILP, Intestinal lamina propria; MLN, mesenteric lymph nodes; PP, Peyer’s patches

The ELISA results revealed that the levels of Th1 cytokine (IFN-γ) and Th2 cytokine (IL-4) in the five groups of mice were not statistically different before immunization (*P* > 0.05). However, the levels of IFN-γ and IL-4 in the two groups of mice immunized with NC8-Tsgal and NC8-Tsgal + α-lactose were clearly higher than the levels in the three control group at 2, 4 and 6 weeks after immunization (*P* < 0.0001). Moreover, the levels of these two cytokines in the two groups of Tsgal-immunized mice continued to be elevated at 1 week after larval challenge, and these levels were maintained to the end of this experiment (5 weeks after infection) (Fig. 6). Nevertheless, no distinct difference in IFN-γ and IL-4 levels was observed between the two Tsgal immunization groups after vaccination and challenge (*P ˃* 0.05). These results demonstrated that immunization of mice with rTsgal triggered the mixed Th1/Th2 responses, that lactose administration had no obvious impact on the cellular immune response of immunized mice and that oral rTsgal immunization produced both the systemic (spleen) and gut mucosal local (MLN, PP and ILP) cellular immune responses.

### Immune protection of NC8-Tsgal immunization

**Fig. 7:**
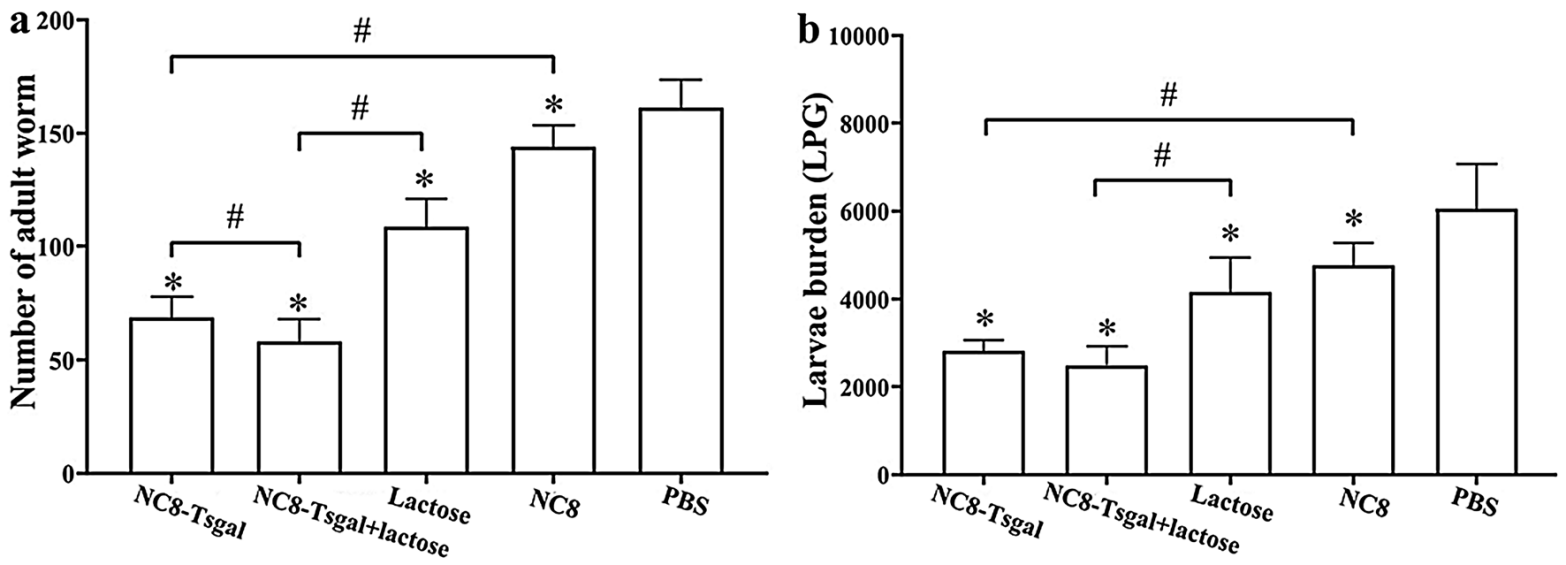
Immune protection elicited by vaccination with recombinant *L. plantarum* NC8 following challenge with 300 *T. spiralis* larvae in a murine model. **a** Intestinal AW burdens, **b** ML burden. The data are shown as the mean worm burden ± SD of 10 animals per group. Asterisk (*)indicates a significant difference at *P* < 0.05 compared to the PBS groups. Hashtag (#) indicates a significant difference at * P* < 0.05 compared between two vaccination groups (one-way ANOVA with Dunnett’s T3 test). LPG, Larvae per gram

Immune protection against *T. spiralis* larval challenge infection was investigated in all vaccinated mice. Compared to the PBS group, the mice vaccinated with NC8-Tsgal, NC8-Tsgal + lactose, lactose and empty NC8 exhibited a 57.28%, 63.92%, 32.61% and 10.73% reduction of intestinal AW burdens, respectively (Fig. 7a). Intestinal AW burdens of the NC8-Tsgal + lactose group were statistically lower than those of the NC8-Tsgal, lactose and empty NC8 groups (*P* < 0.05). Moreover, immunization of mice with NC8-Tsgal, NC8-Tsgal + lactose, lactose and empty NC8 produced a 53.30%, 58.77%, 31.28% and 21.17% reduction of ML burdens at 35 dpi, respectively (Fig. 7b). The ML burdens of mice vaccinated with NC8-Tsgal or NC8-Tsgal + lactose were clearly lower than those of empty NC8 or lactose alone groups (*P* < 0.0001). Additionally, the vaccination of mice with only empty NC8 or lactose alone also showed a partial reduction of intestinal AW and ML compared to the PBS group (*P* < 0.0001). These results demonstrated that vaccination of mice with NC8-Tsgal or NC8Tsgal + lactose elicited an immune protection against *T. spiralis* challenge infection.

### Histopathological changes in intestine and skeletal muscles in immunized mice

Histopathological changes in the intestinal and skeletal muscles of infected mice were investigated at 7 and 35 dpi, respectively. Following *T. spiralis* larval challenge, mild intestinal mucosal inflammation and nearly normal intestinal villi were observed in vaccinated mice (Additional file 1: Figures S2, S3). The width of the enteral villus of the four groups of vaccinated mice was distinctly lower than that of the PBS control group (*P* < 0.0001) (Additional file 1: Fig. S4a). The number of goblet cells of the four groups of vaccinated mice was also overtly lower than that of the PBS group (*P* < 0.0001) (Additional file 1: Fig. S4b). Moreover, the width of the enteral villus was significantly less and the number of goblet cells of mice immunized with NC8-Tsgal or NC8-Tsgal + lactose was lower significantly lower than those of the empty NC8-only and lactose-only groups (*P* < 0.01). These results demonstrated that vaccination of mice with NC8-Tsgal or NC8-Tsgal lactose significantly hindered larval invasion of gut mucosa and ameliorated intestinal inflammation.

The quantitative PCR results showed that the Muc2 transcription level of the four groups of vaccinated groups at 7 dpi was also apparently lower than that of the PBS groups (*P* < 0.0001) (Additional file 1: Fig. S4c). Furthermore, Muc2 transcription level of the NC8-Tsgal group was distinctly lower than that of the lactose-only group (*t* = 7.836, *P* < 0.05), and the Muc2 transcription level of the NC8-Tsgal + lactose group was also clearly lower than that of the empty NC8 group (*t* = 3.071, *P* < 0.05). These results demonstrated that vaccination of mice with NC8-Tsgal or NC8-Tsgal + lactose significantly hindered larval invasion of the gut mucosa, ameliorated the intestinal inflammatory reaction and decreased Muc2 expression.

The results of HE staining of muscle sections of infected mice revealed that the numbers of encapsulated *T. spiralis* larvae of the four vaccinated groups at 35 dpi were distinctly lower than that of the PBS control groups (*P* < 0.0001). Additionally, the number of inflammatory infiltrative cells around the encapsulated larvae of the four vaccination groups was significantly lower than that of the PBS groups (*P* < 0.0001) (Additional file 1: Figures S5, S6). The number of inflammatory cells in the NC8-Tsgal group was notably lower than that of lactose-only group (*t* = -3.103, *P* < 0.05), and the number of inflammatory cells in the NC8-Tsgal + lactose group was also distinctly lower than that in the empty NC8 group (*t* = -3.139, *P* < 0.05). The number of encapsulated larvae in the NC8-Tsgal group was clearly lower than that in the lactose-only group (*t* = - 8.000, *P* < 0.05), and the number of encapsulated larvae in the NC8-Tsgal + lactose group was significantly lower than that in the empty NC8 group (*t* = - 6.342, *P* < 0.05). These results indicated that the vaccination of mice with recombinant *L. plantarum* NC8-Tsgal significantly reduced the number of encapsulated larvae and modulated the inflammatory reaction of the skeletal muscle tissues.

## Discussion

*Trichinella* infection results from eating infected meat. Intestinal mucosal immunity elicited by immunization should block parasite penetration and dislodge and expel intestinal parasites from the gut [18]. Oral immunization is the more appropriate way to induce lasting gut mucosal immune response [42, 54]. *Lactobacillus plantarum* is a lactic acid bacterium capable of colonizing in the gut. *Lactobacillus plantarum *strain NC8 is found in silage and is widely used as a host bacterium to express foreign proteins [21]. It has also been used as a carrier for the construction of immune protective vaccines because of its probiotic effect [24]. Vaccination of chicks with recombinant *L. plantarum* NC8 has been shown to improve humoral and cellular immunity and enhance the resistance to *Eimeria tenella* infection [55]. Hence, in the present study we used *L. plantarum* NC8 to construct recombinant Tsgal vaccine. Additionally, the surface of the pgsA' display module is effective for anchored foreign protein expression, so the plasmid pSIP409-pgsA′ was applied to construct the recombinant Tsgal plasmid to assure the rTsgal expression.

Galectins are characterized by a unique carbohydrate-binding functional domain sequence motif binding to β-galactoside, and this binding can be inhibited by oligosaccharide. The parasite-derived galectins are involved in parasite adhesion and the invasion of host cells [56]. Recombinant *Haemonchus contoutus* galectins have been proved to be a potential vaccine target against challenge infection [57]. Our previous studies showed that rTsgal promotes the in vitro larval invasion of IECs and that anti-rTsgal serum and α-lactose inhibit larval invasion [23]. In order to assess the protective effect of rTsgal and α-lactose on *T. spiralis* larval challenge, in this study we performed experiments using recombinant *L. plantarum* NC8-Tsgal/α-lactose to inoculate the mice.

The results of the current study demonstrated that oral immunization of mice with NC8-Tsgal elicited a Th1/Th2 mixed immune response to Tsgal. T cells and immunoglobulins are important mediators of rapid worm expulsion from the host’s gut [58]. T helper cells largely determine the type of immune response and can be divided into Th1 cells and Th2 cells according to differences in the cytokine expression profiles. The balance between the two types depends on genetic and environmental factors. Th1 cells can secrete IL-2 and IFN-γ, enhance the ability of phagocytes to phagocytose pathogens and promote the production of IgG, CD40 ligand (CD40L) on the surface of Th1 cells, thereby promoting class switching in B cells to generate IgG2a antibodies. Th2 cells secrete IL-4, which can promote the proliferation and differentiation of B lymphocytes and stimulate the production of IgG1 and IgE. Th2 cells have been found to participate in the immune response against intestinal nematode infection, which is beneficial to the repair or prevention of tissue damage caused by helminths [59]. IgG is the main immune force against pathogen infection, and it is also one of the main antibodies produced by the second humoral immune response. The results of this study also showed that serum-specific antibody levels further increased and were maintained at the higher levels in the two groups of immunized mice after larval challenge, suggesting that oral vaccination with NC8-Tsgal significantly enhanced the antigen-specific humoral immunity [24]. Furthermore, the levels of serum-specific IgG and IgA and of intestinal sIgA in the two groups of mice immunized with rTsgal were not statistically different after vaccination and challenge, indicating that lactose inoculation did not affect or enhance the humoral immune response of NC8-Tsgal vaccination.

Intestinal sIgA plays a crucial role in the intestinal mucosal immune response and is structurally resistant to chemical degradation of exogenous enzymes. Most infectious pathogens invade the host through mucosal surfaces, and sIgA is the first natural defense barrier at these surfaces [60]. sIgA plays a vital role in mucosal defense and might impede parasite penetration into gut epithelium [33, 44]. sIgA as an agent against surface antigens of intestinal *T. spiralis* stages (e.g. IIL and AW) has been reported to accelerate worm expulsion from the gut [16], and the passive transfer of anti-*Trichinella* IgA-mediated *Trichinella* expulsion from murine intestine after challenge [61]. Moreover, the immune protection induced by NC8-Tsgal immunization might be due to the formation of an anti-Tsgal antibody immune complex at the worm anterior which physically blocks the direct contact between IIL and gut epithelium and subsequently blocks the penetration of larvae into gut mucosa, thereby preventing further intestinal larval development [16]. Oral immunization with NC8-Tsgal induced higher levels of mucosal sIgA, including total sIgA and *Trichinella*-specific sIgA, indicating that NC8-Tsgal strongly elicited mucosal sIgA secretion. *Lactobacillus* can colonize the intestinal region and induce IgA secretion. *Lactobacillus* has the ability to modulate dendritic cell properties, for example, by inducing B cells to produce IgA [62]. Our observations further indicate that vaccinated mice generated Tsgal-specific enteral sIgA. sIgA is Th2 dependent; in particular, IL-4 is the main cytokine which enhances IgA response, suggesting that high levels of IL-4 enhance gut sIgA response [30].

Helminth infections are characterized by a biased Th2-type immune response, and it has been shown that gastrointestinal worms trigger regulatory pathways to limit Th1-type responses [63]. IL-4 stimulates the production of IgE, mast cells and mucus, enhances intestinal smooth muscle contractility and intestinal epithelial fluid secretion, stimulates CD4 + T cells to differentiate into Th2 cells, promotes IFN-γ secretion and inhibits type 2 cytokine secretion [64]. Our results also confirmed that 2 weeks after the first immunization, the levels of IL-4 and IFN-γ in NC8-Tsgal immunized mice increased significantly, and continued to increase after challenge. Th2-type immune responses are essential for intestinal nematode infection and mainly manifest as mast cell and goblet cell hyperplasia, increased mucus, increased soluble mediators (e.g. IL-4, IL-5, IL-9, IL-13 and histamine) and the production of antibodies (IgG1 and IgE) [59]. Goblet cells are intestinal epithelial mucus-secreting cells that promote worm expulsion from the gut by secreting mucus; the number of goblet cells is closely correlated with the severity of *T. spiralis* infection. In one study, obvious goblet cell proliferation and increased mucin secretion were considered to demonstrate serious *T. spiralis* infection [65]. Histamine is mainly secreted by mast cells; this molecules can induce smooth muscle contract and promote intestinal peristalsis and worm expulsion [66]. The production of IL-9 and its binding to its receptor in muscles also promote intestinal muscle hypercontractility and accelerated worm expulsion from the gut in *T. spiralis* infection [67]. In the present study, intestinal villus width, goblet cell number and Muc2 expression level of the mice immunized with NC8-Tsgal or NC8-Tsgal + lactose were significantly lower than that in the only empty NC8-Tsgal and lactose-only groups, demonstrating that vaccination of mice with NC8-Tsgal or NC8-Tsgal + lactose significantly hindered larval invasion of the gut mucosa, ameliorated intestinal inflammation and relieved the infection. Moreover, the histamine level in the NC8-Tsgal, NC8-Tsgal + α-lactose, and empty NC8 groups was significantly increased at 4 and 6 weeks after the immunization, continued to be elevated at 1 week after challenge and regressed at 5 weeks after infection, suggesting that oral immunization of mice with recombinant NC8-Tsgal or empty NC8 triggered an intestinal mucosal immune response and histamine secretion, which in turn promoted worm expulsion from the gut. Furthermore, the ELISA assay of cytokine response in the current study revealed that cells from the spleen, MLN, PP and ILP were stimulated by the purified rTsgal. Although rTsgal was retrieved with High Capacity Endotoxin Removal Resin, it still might contain a little of the bacterial endotoxin (lipopolysaccharide [LPS]) after treatment. The LPS or same Ni–NTA fraction but from an expression of the same empty pQE-80L plasmid in BL21 (DE3) should be used as a control for cellular stimulation in future studies.

The results of the challenge infection showed that oral vaccination of mice with NC8-Tsgal and NC8-Tsgal + lactose resulted in a significant immune protection against *T. spiralis* challenge, as demonstrated by a 57.28% and 63.92% reduction of intestinal AW burden, respectively, and a 53.30% and 58.77% reduction of ML burden, respectively. It is interesting that oral inoculation with lactose only also produced a 32.61% reduction in the AW burden and a 31.28% reduction in the ML burden. As a surface protein of *T. spiralis* intestinal stages, Tsgal comes into direct contact with the host's gut mucosal epithelium, specifically binding to the galectin ligands of the IECs, thereby promoting the larval invasion of IECs [23]. Lactose might interrupt the interaction between Tsgal and its ligands in the IECs and, as a result, impede the larval invasion of gut mucosa and facilitate worm expulsion from the gut, therefore reducing intestinal AW burdens and alleviating the infection. The results of HE and PAS staining of intestinal and muscle sections also revealed that the lactose-only administration decreased the worm burden and ameliorated the inflammatory reaction of the gut and muscle tissues. These results suggested that binding of Tsgal with IECs might be significantly reduced by competition with anti-Tsgal antibody and with lactose. The sugar that binds to the carbohydrate-binding domain of Tsgal might also limit its engagement of T cell immunoglobulin and Muc2 receptors [68]. Previous studies have shown that an oligosaccharide (mannose) might bind to the C-type lectin on the cuticle surface of *T. spiralis* and hinder its interactions with the ligands on the host’s IECs, which in turn might prevent larval penetration [69]. Recent studies have also revealed that β-glucans trigger *T. spiralis* worm expulsion from the gut via the mucus layer independently of type-2 immunity [53]. Taken together, these results demonstrated that appropriate sugars might be regarded as a convenient and prospective adjuvant agent of anti-*Trichinella* vaccines to impede larval invasion and enhance worm dislodgment at the early stage of *T. spiralis* exposure and infection.

Additionally, galectin-1-like proteins (TsGal-1-like) have been isolated from *T. spiralis* ML; the ES proteins which had a lactose-specific carbohydrate-recognition domain were recognized by anti-galectin-1 antibodies on western blotting. This TsGal-1-like isolate induced dendritic cells with tolerogenic properties and, hence, the capacity to polarize T cell response towards a regulatory type, as demonstrated by a significantly increased percentage of CD4+CD25+Foxp3+ regulatory T cells and a significantly increased expression of IL-10 and tumor growth factor beta (TGF-β) within this cell population, with maintenance of immune homeostasis [70]. Previous studies revealed that some homologs of galectin-9 isolated from canine intestinal nematode *Toxascaris leonina* promote significantly increased levels of TGF-β and IL-10, and these regulatory cytokines may ameliorate intestinal inflammation [71]. Moreover, following oral antigen administration, intestinal epithelial cells and microbiota possibly condition dendritic cells toward a tolerogenic phenotype that induces Treg via expression of several mediators (e.g. IL-10, and TGF-β) [72]. In the present study, oral vaccination of mice with NC8-Tsgal/NC8-Tsgal + lactose might also have regulated the gut microbiota, induced regulatory T cells, promoted the production of IL-10 and TGF-β and, as a result, relieved intestinal inflammation. Therefore, the levels of the cytokines TGF-β and IL-10 following oral Tsgal vaccination should be evaluated in future research.

## Conclusions

Recombinant *L. plantarum* NC8-Tsgal was constructed in the current study. rTsgal protein was expressed on the surface of recombinant NC8-Tsgal. Oral vaccination of mice with recombinant NC8-Tsgal vaccine elicited a systemic mixed Th1/Th2 immunity as well as local gut mucosal response, and an obvious immune protection against *T. spiralis* challenge. The results indicated that recombinant NC8-Tsgal vaccine is a promising strategy for control of *Trichinella* infection in food animals. Moreover, appropriate sugars might be a convenient and prospective adjuvant agent of anti-*Trichinella* vaccines to impede *T. spiralis* larval invasion at early infection stage.

## Supplementary Information

**Additional file 1:** **Figure S1.** Biological characteristics of recombinant *L. plantarum* NC8-Tsgal. **a** growth curve of recombinant *L. plantarum* NC8-Tsgal and normal *L. plantarum *without pSIP409-pgsA′-Tsgal as the control. **b** tolerance of NC8-Tsgal in acid condition. *P < 0.05 compared to the number of NC8-Tsgal at pH 1.0-4.0 (one-way ANOVA with LSD test). **Figure S2.** Enteral histopathological changes in immunized mice at 7 days after challenge infection with 300 *T.* *spiralis* larvae. **Figure S3.** PAS staining of intestinal sections from immunized mice at 7 days after challenge infection with 300 *T.* *spiralis* larvae. **Figure S4.** Enteral pathological changes in vaccinated mice at 7 days after *T. spiralis *larval challenge. **a** Intestinal villus width at 7 dpi. **b** Number of intestinal goblet cells at 7 dpi. **c **Relative mucin 2 mRNA expression level. *P < 0.0001 compared to the PBS group (one-way ANOVA with LSD test); ^#^*P* < 0.05 compared between two vaccination groups (Student’s t-test). **Figure S5.** Muscle pathological changes in immunized mice at 35 days after *T. spiralis* challenge infection. **Figure S6** Muscle pathological changes in vaccinated mice at 35 days after *T. spiralis *larval challenge.** a **Number of encapsulated muscle larvae in different vaccination groups. **b **Number of inflammatory cells around encapsulated larvae in different vaccination groups. *P < 0.05 compared to the PBS groups (one-way ANOVA with LSD test). ^#^*P* < 0.05 compared between two vaccination groups (Student’s t-test).

## Data Availability

The data supporting the conclusions of this article have been included within the article.

## Abbreviations

AEC: 3-Amino-9-ethylcarbazole
AW: Adult worms
BSA: Bovine serum albumin
ES: Excretory/secretory
HE: Hematoxylin and eosin
HRP: Horseradish peroxidase
IECs: Intestinal epithelium cells
IFN: Interferon
IFT: Immunofluorescence test
IIL: Intestinal infectious larvae
IL: Interleukin
ILP: Intestinal lamina propria
IPTG: Isopropyl β-d-1-thiogalactopyranoside
LAB: Lactic acid bacteria
LPS: Lipopolysaccharide
ML: Muscle larvae
MLN: Mesenteric lymph nodes
Muc2: Mucin 2
NBL: Newborn larvae
PAS: Periodic acid-schiff stain
PBS: Phosphate-bufered saline
PP: Peyer’s patches
TBST: Tris-buffered saline containing Tween
Tsgal: *Trichinella spiralis* galectin
