# Supplementary material for: Oral immunization of mice with recombinant Lactobacillus plantarum expressing a Trichinella spiralis galectin induces an immune protection against larval challenge
Source: Parasit Vectors. 2022 Dec 20;15:475. doi: 10.1186/s13071-022-05597-w (PMC9764493; doi:10.1186/s13071-022-05597-w)
Supplement: Supplementary file 1 — Additional file 1: Figure S1. Biological characteristics of recombinant L. plantarum NC8-Tsgal. a growth curve of recombinant L. plantarum NC8-Tsgal and normal L. plantarum without pSIP409-pgsA′-Tsgal as the control. b tolerance of NC8-Tsgal in acid condition. *P < 0.05 compared to the number of NC8-Tsgal at pH 1.0-4.0 (one-way ANOVA with LSD test). Figure S2. Enteral histopathological changes in immunized mice at 7 days after challenge infection with 300 T. spiralis larvae. Figure S3. PAS staining of intestinal sections from immunized mice at 7 days after challenge infection with 300 T. spiralis larvae. Figure S4. Enteral pathological changes in vaccinated mice at 7 days after T. spiralis larval challenge. a Intestinal villus width at 7 dpi. b Number of intestinal goblet cells at 7 dpi. c Relative mucin 2 mRNA expression level. *P < 0.0001 compared to the PBS group (one-way ANOVA with LSD test); #P < 0.05 compared between two vaccination groups (Student’s t-test). Figure S5. Muscle pathological changes in immunized mice at 35 days after T. spiralis challenge infection. Figure S6 Muscle pathological changes in vaccinated mice at 35 days after T. spiralis larval challenge. a Number of encapsulated muscle larvae in different vaccination groups. b Number of inflammatory cells around encapsulated larvae in different vaccination groups. *P < 0.05 compared to the PBS groups (one-way ANOVA with LSD test). #P < 0.05 compared between two vaccination groups (Student’s t-test). [file 13071_2022_5597_MOESM1_ESM.doc]

**Additional file 1**


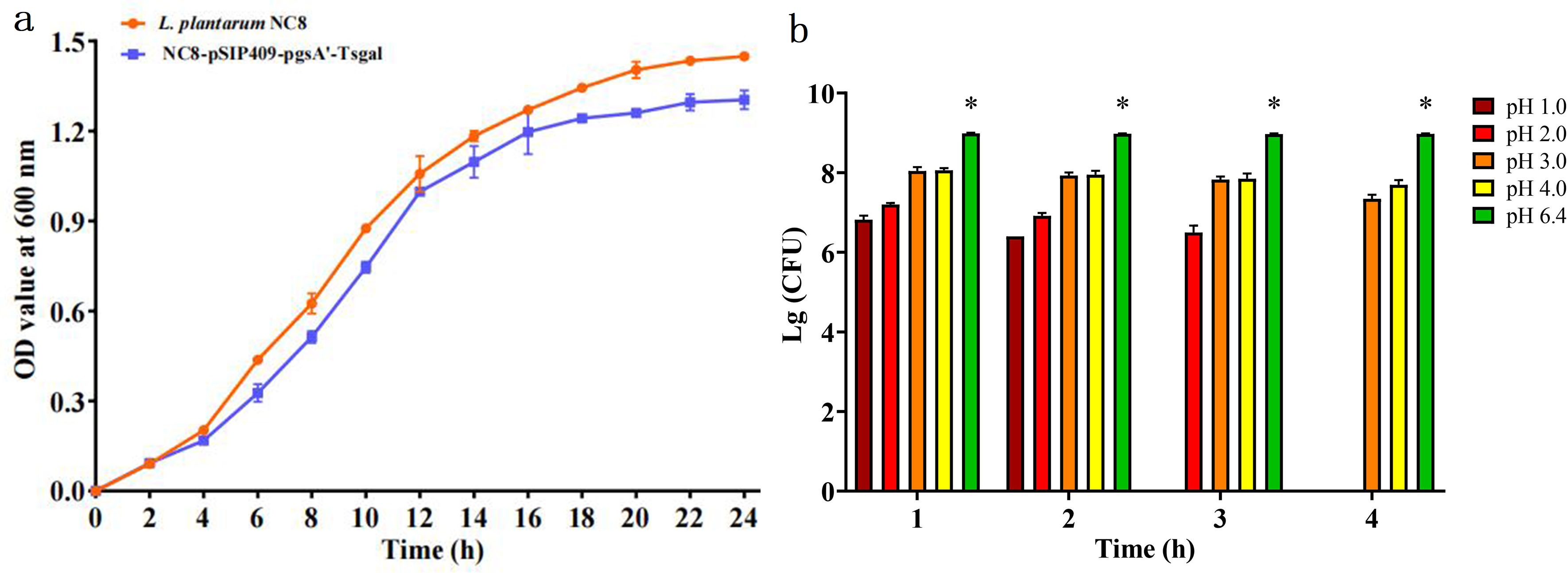


**Figure S1. Biological characteristics of recombinant *L. plantarum*** NC8-Tsgal**. A:** growth curve of recombinant *L. plantarum* NC8-Tsgal and normal *L. plantarum* without pSIP409-pgsA′-Tsgal as the control. **B:** tolerance of NC8-Tsgal in acid condition. As the number of recombinant *L. plantarum* NC8-Tsgal bacteria was too large, the data were compared using logarithm transform. Results are expressed as mean ± SD of three replicates. **P* < 0.05 compared to the number of NC8-Tsgal at pH 1.0-4.0 (one-way ANOVA with LSD test).

**
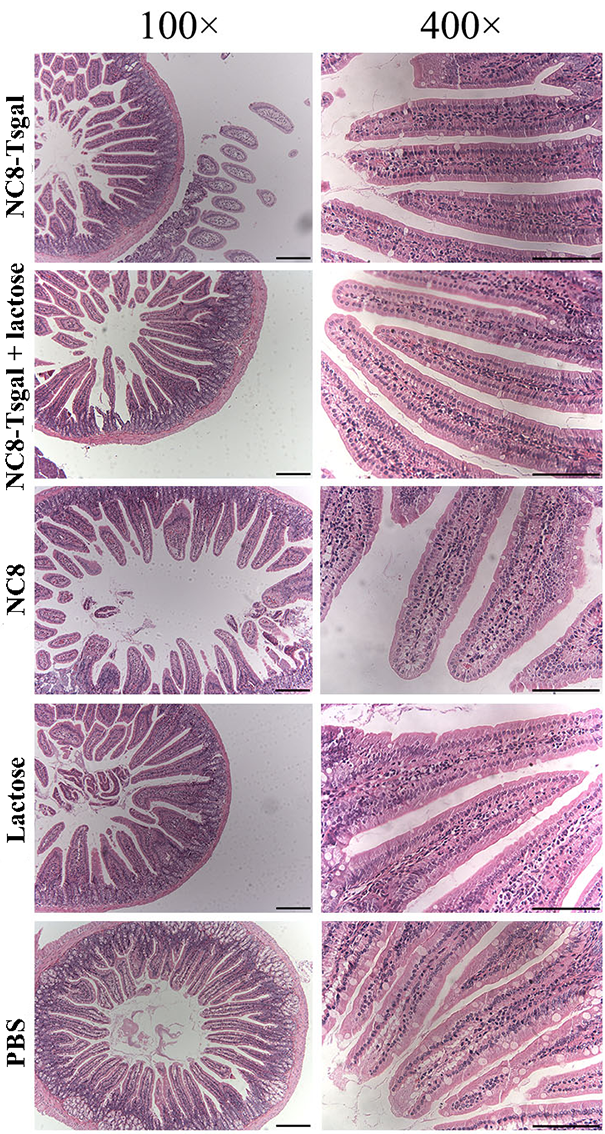
**

**Figure S2. Enteral histopathological changes in immunized mice at 7 days after challenge infection with 300 *T. spiralis* larvae.** Intestinal section was stained with haematoxylin and eosin (HE) and examined under light microscopy. Intestinal pathological changes from four groups of vaccinated mice were obviously alleviated compared to the PBS group. The seriously intestinal inflammation and widen intestinal villi were observed in intestinal section of the PBS control groups. Scale bars = 200 μm.


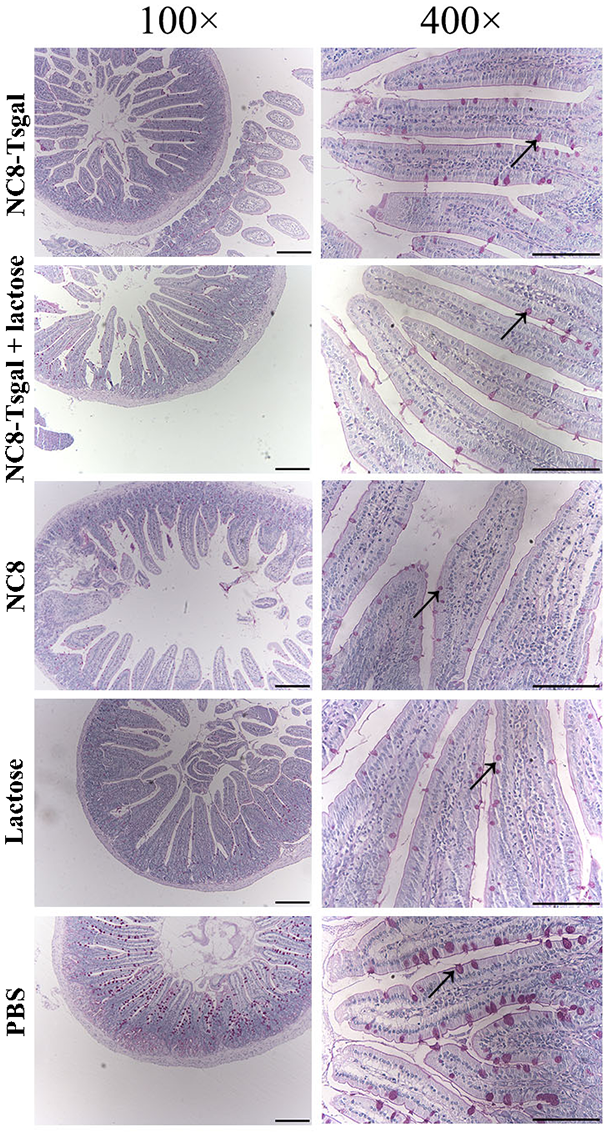


**Figure S3 PAS staining of intestinal sections from immunized mice at 7 days after challenge infection with 300 *T. spiralis* larvae.** The numbers of goblet cells of four groups of vaccinated mice were obviously reduced compared to the PBS group. Goblet cells were marked with solid arrows. Scale bars = 200 μm.

**
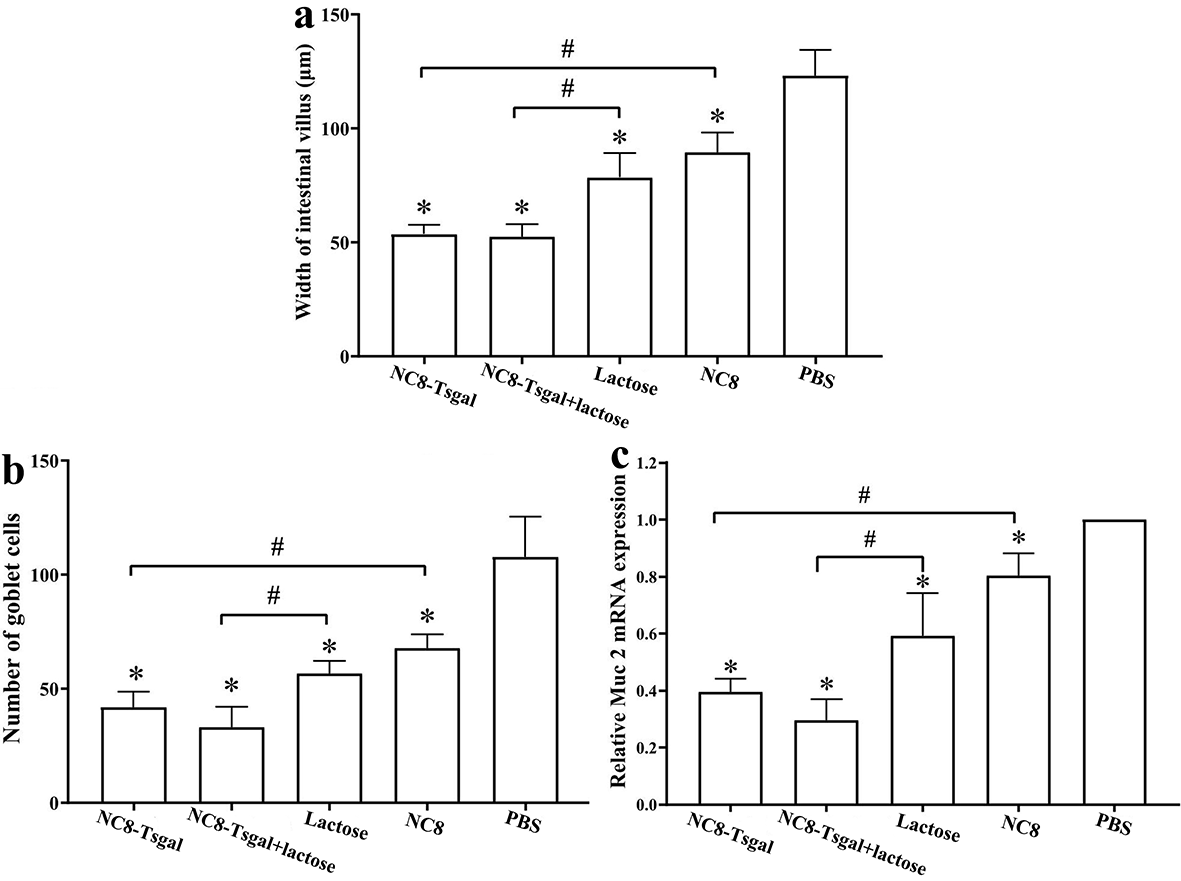
**

**Figure S4 Enteral pathological changes in vaccinated mice at 7 days after *T. spiralis* larval challenge.** **a** Intestinal villus width at 7 dpi. **b** Number of intestinal goblet cells at 7 dpi. The data of intestinal villus width and goblet cells are presented as the mean ± SD of three animals per group. **c** Relative mucin 2 mRNA expression level in gut epithelium of five animals per group, all samples were assayed in triplicate, and data show the mean ± SD of five animals per group. **P* < 0.0001 compared to the PBS group (one-way ANOVA with LSD test); ^#^*P* < 0.05 compared between two vaccination groups (Student’s t-test).

**
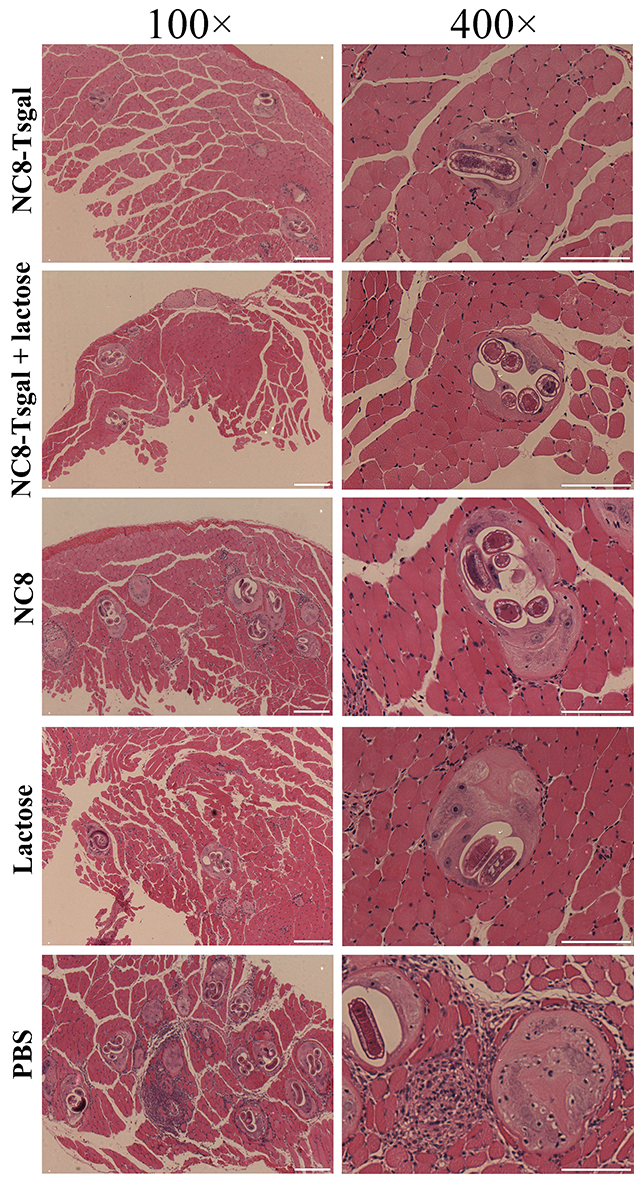
**

**Figure S5. Muscle pathological changes in immunized mice at 35 days after *T. spiralis* challenge infection.** Mild inflammatory reaction and less encapsulated larvae were observed in muscle section of two groups of mice immunized with NC8-Tsgal or NC8-Tsgal+lactose. Scale bars = 200 μm.


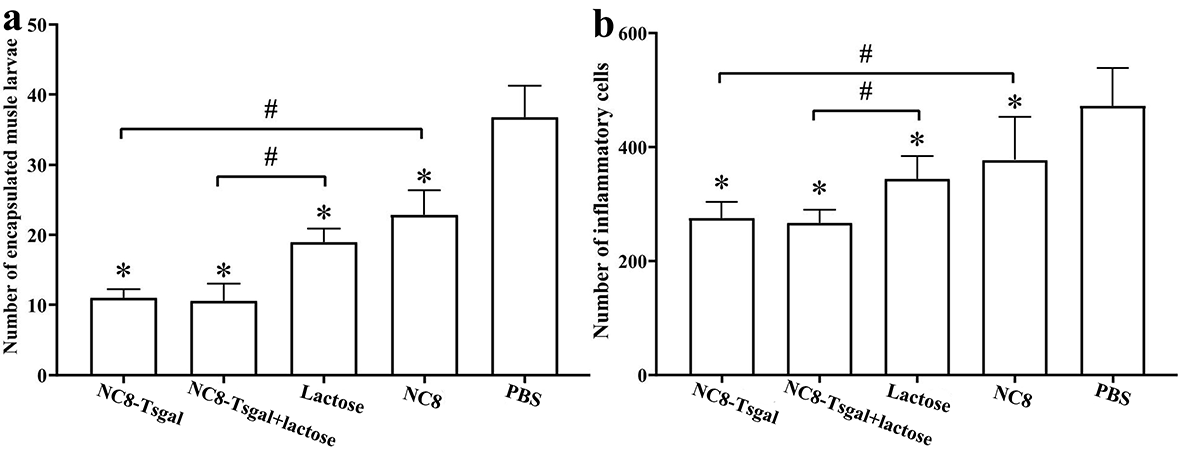


**Figure S6 Muscle pathological changes in vaccinated mice at 35 days after *T. spiralis* larval challenge. a** Number of encapsulated muscle larvae in different vaccination groups. **b** Number of inflammatory cells around encapsulated larvae per field (×400) in different vaccination groups. Data show the mean ± SD of three animals per group. * *P* < 0.05 compared to the PBS groups (one-way ANOVA with LSD test). ^#^*P* < 0.05 compared between two vaccination groups (Student’s t-test).
